# Radiation-induced cardiac side-effects: The lung as target for interacting damage and intervention

**DOI:** 10.3389/fonc.2022.931023

**Published:** 2022-07-22

**Authors:** Julia Wiedemann, Robert P. Coppes, Peter van Luijk

**Affiliations:** ^1^ Department of Biomedical Sciences of Cells and Systems, University Medical Center Groningen, University of Groningen, Groningen, Netherlands; ^2^ Department of Radiation Oncology, University Medical Center Groningen, University of Groningen, Groningen, Netherlands

**Keywords:** lung, heart, cardiotoxicity, radiation, pulmonary hypertension, vascular remodeling

## Abstract

Radiotherapy is part of the treatment for many thoracic cancers. During this treatment heart and lung tissue can often receive considerable doses of radiation. Doses to the heart can potentially lead to cardiac effects such as pericarditis and myocardial fibrosis. Common side effects after lung irradiation are pneumonitis and pulmonary fibrosis. It has also been shown that lung irradiation has effects on cardiac function. In a rat model lung irradiation caused remodeling of the pulmonary vasculature increasing resistance of the pulmonary vascular bed, leading to enhanced pulmonary artery pressure, right ventricle hypertrophy and reduced right ventricle performance. Even more pronounced effects are observed when both, lung and heart are irradiated.

The effects observed after lung irradiation show striking similarities with symptoms of pulmonary arterial hypertension. In particular, the vascular remodeling in lung tissue seems to have similar underlying features. Here, we discuss the similarities and differences of vascular remodeling observed after thoracic irradiation compared to those in pulmonary arterial hypertension patients and research models. We will also assess how this knowledge of similarities could potentially be translated into interventions which would be beneficial for patients treated for thoracic tumors, where dose to lung tissue is often unavoidable.

## Introduction

According to the WHO (world health organization) cancer is one of the leading causes of death worldwide with around 10 million cases in the year 2020. Lung and breast cancer are the two most diagnosed cancer types (1). However, improvement of therapy in the last decades has enhanced survival significantly. Around 50% of all cancer patients receive radiotherapy during their treatment (2), with lung and breast cancer amongst the leading indications (3). When treating thoracic tumors with radiotherapy, heart and/or lung tissue often receive substantial radiation doses.

As survival rates improve, irradiation effects of normal tissue become an increasing concern. Radiation-induced heart diseases (RIHD) are one of the major side effects after thoracic radiotherapy (4). Several recent studies have shown that the risk of cancer patients dying from cardiovascular events is higher than in the general population (5–7). Typical RIHDs that are seen are valvular disease, pericardial disease, conduction abnormalities, cardiomyopathy, and accelerated coronary artery disease (4). Several recent review articles have pointed to radiation-induced endothelial damage in the heart tissue as sign of cardiac injury, followed by the activation of several inflammatory pathways, the release of specific cytokines and immune cell activation which, in combination, finally lead to fibrosis (4, 8, 9). However, previous studies have mostly only investigated direct exposure of the heart or its substructures as a possible cause of RIHD. In this review, we aim to summarize evidence that irradiation of lung tissue also has an influence on the heart, especially on RV function, and that the interplay of effects on heart and lung contribute to radiation-induced cardiac toxicity. Furthermore, we hypothesize that the irradiation effects on lung tissue as well as the resulting cardiac effects show remarkable similarities to features of pulmonary arterial hypertension (PAH). The role of the lung in the development of cardiac side-effects suggests that this may offer novel targets for interventions to prevent such side-effects. Therefore, we aim to show parallels of the effects of lung irradiation with features observed in pulmonary arterial hypertension, and discuss possible subsequent treatment options.

## Radiation effects on lung tissue contribute to cardiotoxicity

It is well known and reported that lung irradiation induces RILI (radiation induced lung injury) which is often divided in early acute toxicity manifesting as radiation pneumonitis and chronic toxicity resulting in pulmonary fibrosis (10–12). As primary mechanisms of damage, direct DNA damage and the generation of reactive oxygen species (ROS) are described. Radiation- induced apoptotic cell death of epithelial and endothelial cells, together with the effects of ROS, contribute to an inflammatory state leading to radiation pneumonitis (11). Cytokines and growth factors released during the inflammatory state enhance collagen production in fibroblasts, and finally lead to late phase pulmonary fibrosis (12).

However, in addition to these often-described effects of lung irradiation, vascular damage and vascular remodeling also occurs in the time frame of several weeks after irradiation. The loss of vascular endothelial cells and the consequent loss of barrier function is accompanied by the thickening of vessel walls, occlusion of small vessels and perivascular edema (13, 14). Vascular remodeling was observed after whole thorax irradiation, in several studies performed in small animals (15, 16). In addition, those studies report enhanced vascular resistance in the lung (15). Such increased resistance in the pulmonary vasculature leads to increased pulmonary artery pressure, which in turn can induce or worsen right ventricle remodeling (17) followed by a reduction of right ventricular performance. **Table 1** summarizes the experiments performed in animal models providing evidence for this. Gosh et al. (16) showed that beside an increased vascular resistance and reduced vessel density, right ventricular hypertrophy could also be observed after whole thorax irradiation of rats with 10 Gy. Although the heart was included in the irradiation field, the authors speculate that lung irradiation may influence right-ventricle function. Testing this hypothesis requires precise irradiation of defined lung volumes, excluding the heart, which is challenging in small animals like rats and mice. Dogs were used in a study in the 1990s to achieve this. This study proposed that pulmonary hypertension is secondary to lung irradiation and is most likely followed by right ventricle hypertrophy (18). A study using high-precision proton irradiation to enable precise irradiation of different lung volumes showed that the irradiation of 75% of the lungs with 17 Gy, while shielding the heart, leads to significant increases in pulmonary artery and right ventricle pressure in combination with right ventricle hypertrophy and vascular remodeling in the lung (19). In the same study, irradiation of the whole lung was shown to lead to pronounced pulmonary dysfunction and vascular remodeling in the absence of major parenchymal remodeling. This highlights that these pulmonary vascular and right ventricle effects can occur at lower doses than parenchymal remodeling, making it a potentially relevant side effect that may be occurring in patients after radiotherapy. Moreover, in the same model, combined irradiation of lung and heart led to a stronger effect, which was explained by the interplay of the two organs in the cardiopulmonary system (20). Vascular remodeling in the lung tissue leads to enhanced resistance and contributes to right ventricular remodeling while the direct irradiation of the heart leads to a reduced diastolic function in the left ventricle. In summary, these preclinical data obtained in dog and rat models show that radiation effects in the lung can lead to cardiac side-effects, even if the heart is not in the irradiation field.

**Table 1:** Summery of preclinical animal studies showing evidence that vascular remodeling after irradiation of the lung can lead to cardiotoxicity.

Reference	Animal model	Irradiation modalities and dose	Irradiation field	Time points
Molthen et al., 2012 (15)	Female WAG/Rij/Cmcr rats	Photons, 10 Gy	Whole thorax	2 month after irradiation
Gosh et al., 2019 (16)	Female WAG/Rij/MCW rats	Photons, 10Gy	Whole thorax	Up to 12 month after irradiation
McChesney-Gilette et al., 1991 (18)	Adult beagle dogs, 1 year old	Photons, 12 Gy	Group I: entire heart and lung Group II: lung only, heart shielded Group III: entire heart+ overlying lung	Up to 24 weeks
Ghobadi et al., 2012 (19)	Adult male albino Wistar rats	Protons, 20Gy	33% lateral lung 50% lateral lung Lung excluding heart Heart+lung	8 weeks
Ghobadi et al., 2012 (20)	Adult male albino Wistar rats	Protons, 20Gy	Heart+ 25% lung Heart+ 50% lung 50% lung	8 weeks

## Pathology of vascular remodeling in PAH and as a result of irradiation

It has long been established in cardiology that vascular remodeling occurs during the development of pulmonary arterial hypertension (21). Pulmonary arterial hypertension is a progressive cardiovascular disease with a high mortality and a strong impact on the quality of life (22). The enhanced pulmonary resistance causes pressure overload of the right side of the heart leading to right-sided heart failure causing potential death. The disease is well-characterized and, although limited, treatment options are available. Interestingly, although the cause differs, the vascular remodeling process leading to right heart failure in PAH shows similarities to those observed after irradiation.

In healthy lungs, vessels consist of three layers: the intima, the media and the adventitia. The intima is the innermost layer consisting of one layer of endothelial cells (ECs). The medial vascular wall is mainly composed of smooth muscle cells (SMCs) which are quiescent in normal lung tissue. The adventitial layer is the outermost vessel layer. It mainly consists of fibroblasts, but also contains immune modulatory cells, resident progenitor cells, endothelial cells, and adrenergic nerves (23).Vascular remodeling observed in PAH is characterized by the thickening of the three layers of the vascular wall (21, 22, 24). Although deposition of extracellular components such as collagen can contribute to the thickening, the main component is hypertrophy and hyperplasia of cells in their corresponding layer. In addition, in peripheral arteries where precursor cells differentiate to SMCs, muscularization can occur. Interestingly, most of these pathological features of vascular remodeling typical for PAH can also be observed in a comparable manner after irradiation. In the following sections this is described in more detail for each layer of the vascular wall.

### The intimal layer

One of the first stages in the development of PAH is apoptosis and dysfunction of endothelial cells. In PAH, endothelial apoptosis is mostly mutation-driven and apoptotic cell loss leads to the disruption of vessel lining and leakage of micro vessels (25). A change in the endothelial cells lining small and larger vessels is also shown in lung tissue after irradiation. A staining with the endothelial cell specific marker HIS52 shows endothelial cell detachment and loss after irradiation leading to a disruption of the endothelial layer (19). In general, apoptosis of endothelial cells is described for irradiation doses above 5 Gy in *in vivo* and *in vitro* experiments (13, 26). For PAH it is described that the remaining endothelial cells are apoptosis resistant and proliferate to regenerate the damage caused by the loss of apoptotic endothelial cells (21, 22, 25). Overshooting proliferation can subsequently lead to massive thickening of the intima and so-called neointima formation. Both are indeed also observed in irradiated lung tissue, contributing to the overall thickening of the vessel walls (19, 27) making it likely that some endothelial cells are resistant to radiation induced apoptosis, and triggering following processes in vascular remodeling (21, 25). Neointima formation and neointimal lesions are features of complex or advanced lesions, while more progressed lesions are called plexiform lesions (28). Neointimal lesions as well as plexiform lesions are characteristic for severe forms of PAH, are mainly observed in a late state of the disease and are often considered as a hall mark of PAH (22, 29). The above-mentioned rat model system of irradiation shows lesions comparable to those advanced lesions including neointima formation (19). The described changes and apoptosis in a subset of endothelial cells in PAH may have a different cause than after irradiation, but this comparison shows that the effects on the vascular wall are quite similar. Therefore, apoptosis and changes in ECs can be a driver of vascular wall thickening and remodeling.

### The media

In PAH the transition of pulmonary arterial smooth muscle cells (PASMCs) to proliferating cells leads to remodeling and thickening of the medial vascular wall, and muscularization in smaller vessels. This often happens in parallel with the above described neointima-formation. Muscularization of small vessels and thickening of the medial layer is also described after lung irradiation in a rat model (19) shown in histological staining as well as in a significant enhanced thickness of the vessel wall together with vessel occlusion. In a rat model of whole thorax irradiation muscularization of a few vessels was observed already at a dose of 10 Gy (15). Proliferation of PASMCs can be caused by mediators released from endothelial cells (30). This is in line with the finding, that *in vitro* irradiation of SMC can lead to inhibition of proliferation and migration, which is used to counteract vascular stenosis (31). This result shows that muscularization, and therefore SMCs proliferation after irradiation, is most likely triggered by factors released from other cells. Senescence of PASMCs and related cytokine release can also play a role in the induction of proliferation (22) and will be discussed later. In addition, hypertrophy of the PASMCs and the enhanced deposition of extracellular matrix contribute significantly to the thickening of the media (29). As such, since thickening of the medial layer and muscularization of small vessels can be observed after irradiation, this is most likely the result of fibroblast activation due to the release of mediators by other cells.

### The adventitia

In an early phase of the restructuring process of the vessel wall in PAH the activation of the fibroblasts in the adventitia and the induction of proliferation and reprogramming processes but also the onset of chemokine and cytokine release occurs. Although not playing a primary role in vessel dysfunction, mild adventitial thickening is known to occur in PAH. Even though not described explicitly in models of lung irradiation, it may be part of the overall observed thickening of the vascular wall in these models (16, 19). However, after coronary brachytherapy, used to counteract neointima formation and restenosis after cardiovascular interventions, a thickening of the adventitial layer has been observed as late adverse effect, even if intima hyperplasia could be inhibited successfully (32, 33). Furthermore, inflammation and activation of the peroxynitrit-PARP-pathway could be observed in the outer vessel wall after gamma irradiation of the carotid arteries (32). The function of the adventitial layer as a hub for inflammation will be discussed later. Adventitial thickening is not amongst the most important mechanisms in PAH. However, this layer of the vascular wall, including several different cell types, plays a crucial role in inflammatory processes and similar effects could be found after irradiation.

Taken together, the main pathological features of PAH - namely the loss of endothelial cells, the thickening of the intimal layer and neointima formation as well as medial thickening and muscularization - are also observed after irradiation. This supports the hypothesis that radiation may induce PAH and might be an overlooked side effect of thoracic radiotherapy. Recognizing PAH as a potential side-effect may open new opportunities to prevent or treat side-effects of thoracic radiotherapy by intervening in mechanisms playing a role in PAH.

## Molecular mechanisms of vascular remodeling

**Figure 1:**
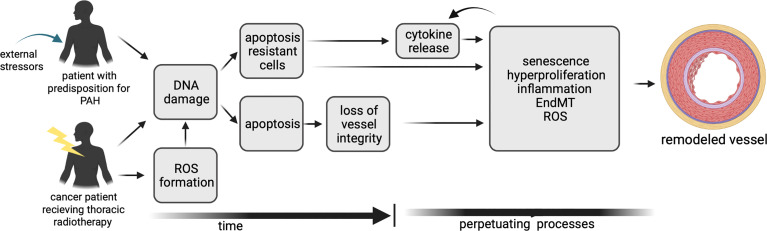
A timeline of the mechanisms contributing to vascular remodeling in PAH and after irradiation, illustrating differences and similarities.

**Figure 2:**
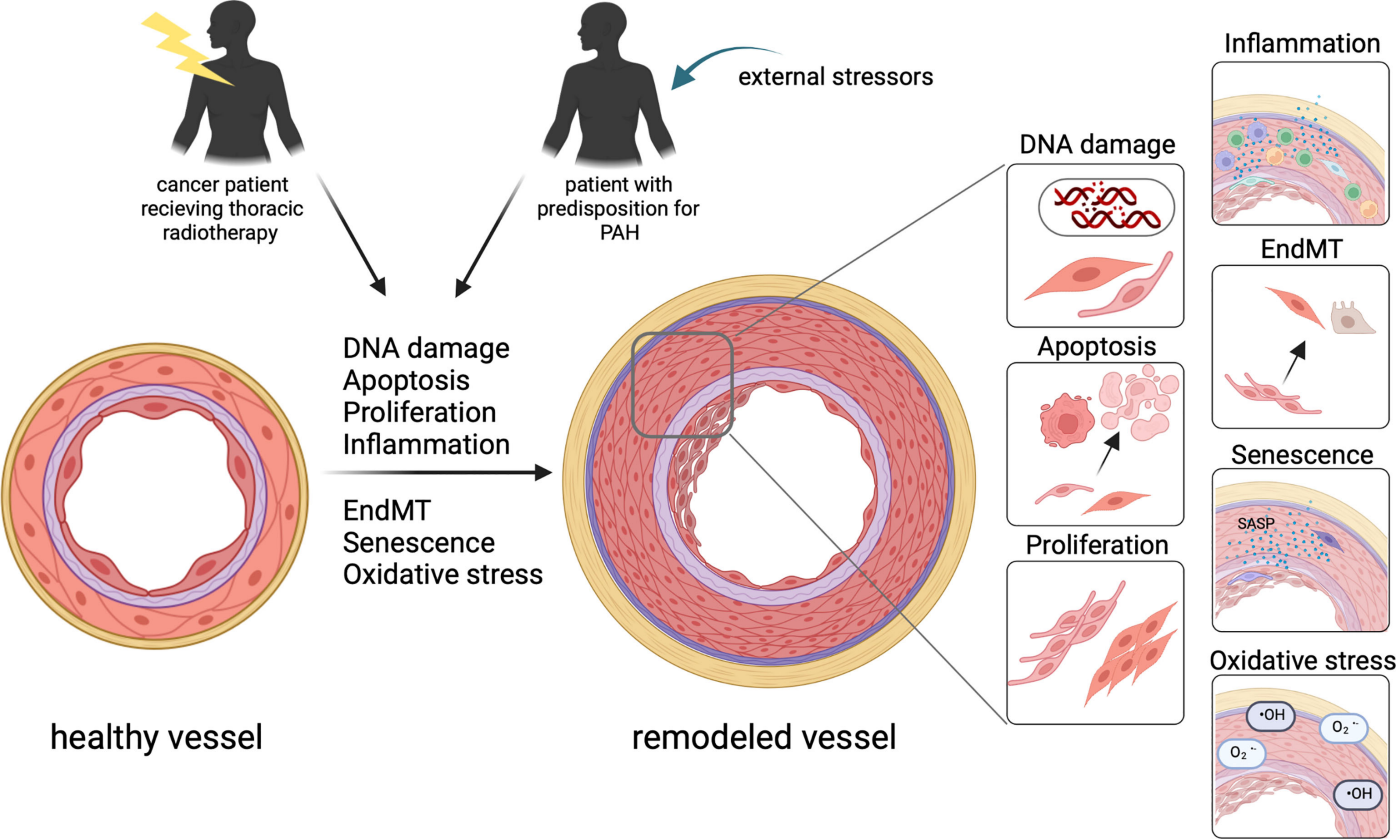
In patients with a predisposition for PAH as well as well as in patients after thoracic radiotherapy, similar mechanisms contribute to vascular remodeling turning a healthy vessel to a vessel with significant thickening of all layers of the vessel wall. This includes DNA damage, apoptosis, proliferation, inflammation, EndMT, senescence and oxidative stress contributing to the remodeling of the vessel.

Although the basic origin is different, there are many parallels in the molecular mechanisms involved in the initiation and progression of vascular remodeling in PAH and after lung irradiation. These include, for example, apoptosis, inflammation, and senescence. Although the chronological order is not completely clear, DNA damage and apoptosis is an initial step (34) (**Figure 1**). in PAH as well as after irradiation. In some of the PAH patients a genetic predisposition is a prerequisite for this (35). DNA damage and apoptosis is followed by the induction of senescence, inflammation and EndMT which are processes occurring most likely in parallel. The radiolysis of water is the main source of ROS after irradiation, occurring during the irradiation process and contributing to an environment favoring other processes such as inflammation (36, 37). In PAH, ROS play a role later in the process, increasing vasoconstriction but also contributing to vascular remodeling. In the following sections the mechanisms will be discussed separately to show parallels (**Figure 2**) playing a role in PAH induction and in vascular remodeling after irradiation.

### DNA damage and apoptosis

Even if the exact timing is unclear, a dysfunctional EC phenotype occurs in parallel with the manifestation of PAH. Indeed, various data support that EC injury and apoptosis are involved in the early steps of PAH development. In an animal model, EC apoptosis has been shown to be a direct trigger for the induction of PAH (34). Apoptosis can have a number of different triggers. Around 10% of PAH patients have a genetic predisposition to develop the disease due to EC apoptosis-prone genotype. They carry heterozygous mutations in the bone morphogenetic protein receptor type 2 (BMPR2) (35), which normally mediates pro-survival signaling in endothelial cells. BMPR2-knock-out experiments in mice (38) showed that the BMPR2 mutation might contribute to the initial step of EC apoptosis in PAH patients. However, unrelated to BMPR2 mutations, endothelial cells of PAH patients are also found to be intrinsically more prone to DNA damage (39). DNA damage can lead directly to apoptosis or initiate the activation of repair pathways. Cells of pulmonary arteries isolated from PAH patients show enhanced expression of the damage marker 53BP1 and γH2AX together with PARP-1 activation as part of the DNA damage response. PARP-1 activation can increase survival and proliferation, playing a role in the progression of PAH. In addition, DNA damage also downregulates BMPR2, decreasing the DNA damage response and further compromising genomic integrity of the cells.

Endothelial cell death has also been described after irradiation. *In vitro* experiments show significant levels of apoptotic cells in primary cultures of ECs derived from bovine adrenal micro vessels after irradiation with a dose of 10 Gy (26). Persistent radiation-induced DNA damage leads to p53 accumulation and caspase activation. EC apoptosis can also be modulated by the sphingomyelin/ceramide pathway. The phospholipid sphingomyelin which is present in the cell membrane is hydrolyzed upon TNF activation after irradiation leading to ceramide-mediated apoptosis of endothelial cells. Enhanced sphingomyelin levels have been shown in patients undergoing high-dose radiotherapy (40). Even if mediated by different pathways, DNA damage leads to apoptosis in PAH as well as after irradiation in endothelial cells and can serve as a critical early step in the initiation of the disease. Regardless of the actual origin, the loss of vessel integrity seems to be a starting point for a similar progression of vascular remodeling in PAH and after irradiation.

### Senescence

Senescence is a process in which cells lose their capability of proliferation and cell growth and are arrested in G0/G1 phase (41). It is characterized by upregulation of senescence-associated genes and significant increase of specific P-galactose activity (42). Typically, senescent cells represent a senescence-associated secretory phenotype (SASP) which can contribute to the initiation and progression of several diseases in which senescence is involved (41). It has been shown that senescence of endothelial cells plays a role in the development and end stage of PAH (42). They show dysfunctional signaling and therefore reduced integrity, contributing to PAH development (38) and progression to an irreversible state (43). Furthermore, the study of van der Feen et al. (43) showed that treatment with the senolytic ABT263 enables the reversal of the hemodynamic and structural changes.

Senescence is also a very well-known phenomenon after irradiation, occurring in different cell types and known to contribute to normal tissue effects. After radiotherapy, healthy cells undergoing senescence contribute to the risk of early and late complications and morbidity as they can contribute to tissue fibrosis and organ dysfunction (44, 45). After lung irradiation senescent fibroblasts contribute with their activated SASP to radiation induced fibrosis (45). It has also been demonstrated *in vitro* and *in vivo* that lung endothelial cells change to a senescent phenotype after exposure to ionizing irradiation (46–49). *In vitro*, enhanced beta-galactosidase activity, typical for senescent cells, as well as an upregulation of senescence-related pathways was described in human pulmonary artery endothelial cells (HPAECs) and in human lung microvasculature endothelial cells (HLMVECs) after exposure to X-ray irradiation (46, 49). Inflammation-related genes, which are also involved in senescence are amongst the most-upregulated genes in these cells after irradiation (46) showing the activation of an SASP and also the relation and interaction of the two mechanisms.

### Inflammation

Another hallmark in initiation, development and progression of PAH is inflammation. Endothelial cells are the source of key mediators for vascular remodeling including growth factors such as FGF-2, angiotensin II (AngII), vasoactive peptides and molecules like nitric oxide (NO), prostaglandin I2 (PGI2), endothelin-1 (ET-1) and pro-inflammatory cytokines like IL-6 and chemokines. Also, secretion of pro-inflammatory adhesion molecules e.g. ICAM1, VCAM and E-selectin from endothelial cells is enhanced. After irradiation-induced cellular damage, endothelial cells acquire a pro-inflammatory phenotype which leads to the release of similar factors as described for ECs in PAH (38). In addition to remodeling and thickening of the intimal layer, overproduction of these mediators also affects fibroblast-like cells and PASMCs in the vascular wall as well as endothelial cells in an autocrine way. In particular, endothelin-1 and IL-6 induce the differentiation from fibroblasts to myofibroblast, which are highly proliferative and proinflammatory. In addition, they produce collagen and other extracellular matrix proteins contributing to pulmonary vascular remodeling. The fibroblasts in the adventitia are suggested to sense vascular damage, leading to the activation of dendritic and progenitor cells resident in the adventitia to release key regulators of vascular remodeling (22, 50). Factors such as GM-CSF, CCL2 and CXCL12 promote the recruitment of leukocytes, which in turn release factors inducing endothelial cell apoptosis or the switch to the pro-inflammatory phenotype of ECs (22, 23, 38, 50). In a later stage of PAH, immune cells, mainly T cells, monocytes and macrophages infiltrate the vascular wall. They regulate the fate of other vascular cell types *via* direct or indirect signaling and are therefore the main driver and regulator for the remodeling of all parts of the vessel walls by triggering activation, migration, differentiation, proliferation and survival of ECs, SMCs and fibroblasts (21).

Interestingly, in a mouse model, lung specific overexpression of IL-6 was sufficient to induce remodeling of the vasculature, mainly muscularization of arteries and neointima formation, leading to enhanced RV systolic pressure and RV hypertrophy (51). Also, in PAH Patients, enhanced serum levels of IL-6 have been observed, underlining the role of IL-6 as an important mediator in PAH (22). Both *in vitro* and *in vivo* experiments demonstrated that irradiation leads to increased IL-6 release from endothelial cells and fibroblasts (52–54).

The described release of inflammation related mediators from different cell types in PAH as well as after irradiation, show that in both cases a pro-inflammatory milieu is established which contributes to the progression of changes in the vascular walls and vascular remodeling. With the radiation-induced release of IL-6 from endothelial cells and fibroblasts, a key mediator and initiator for PAH associated processes is present.

### Endothelial to mesenchymal transition

Another important process in the development of PAH is the transition of activated endothelial cells into cells expressing markers of SMCs or mesenchymal cells (EndMT), explaining the presence of alpha-smooth-muscle-actin positive cells in the media (55, 56). During the process of the transition endothelial cells lose the expression of the endothelial cell marker CD31 and cadherin, gap junctions are lost, the cells dissociate from the basement membrane and start to migrate into the medial layer. During this process they start to express markers such as alpha-smooth muscle actin or vimentin (56). The transformed cells fail to form an intact barrier and are primed for proliferation and migration, potentially contributing to the formation of neointimal lesions (57). In addition, they can acquire a pro-inflammatory phenotype, after which their release of cytokines contributes to the inflammatory milieu in PAH. In PAH, EndMT is induced through the Smad2/3, Erk1/2 and p38MAPK pathways but can also occur in response to inflammatory cytokines like IL-1beta and TNF-alpha.

Interestingly, EndMT is also induced after radiotherapy (58). In normal tissue, radiation-induced EndMT is mainly recognized as a driver for radiation induced pulmonary fibrosis (RIPF). However, it also contributes directly to vascular remodeling in the early phase of RIPF development and is a process occurring prior to mesenchymal transition of alveolar epithelial cells. *In vitro* experiments using human pulmonary microvascular endothelial cells (HPMVECs) showed an upregulation of mesenchymal markers accompanied by a downregulation of EC markers in RNA sequencing data after exposure to ionizing radiation (46). In line with this, EndMT is dependent on TGF-betaR1 and Smad signaling in an *in vitro* model after irradiation (58). This is similar to the activation processes in PAH. In addition, radiation induced EndMT of ECs promotes the transition from fibroblasts to myofibroblasts which also contribute to vascular remodeling (59). In conclusion, EndMT occurs in endothelial cells in PAH, but it is also a well-known irradiation-induced process, not only contributing to radiation induced fibrosis but also to vascular remodeling. This again shows the parallels in both mechanisms.

### Reactive oxygen species and oxidative stress

Reactive oxygen species (ROS) summarizes a group of reactive molecules, including free radicals, superoxide (O_2_−), hydroxyl (•OH−) and hypochlorite (OCl−), but also non-radical species such as hydrogen peroxide (H_2_O_2_) and other peroxides (ROOH) which can oxidize other molecules (60, 61). An imbalance due to enhanced ROS production and reduced production of antioxidants leads to oxidative stress (62). It has been shown in patients as well as in animal models that oxidative stress and increased ROS levels play an important role in the development of PAH (61–64). Oxidative stress contributes to PAH in different ways: It interferes with production of vasodilators like NO and PGI2 from vessels by damaging the endothelial nitric oxide synthase (eNOS) and prostacyclin synthase (PGIS) leading to enhanced vasoconstriction (64). It also promotes vasoconstriction by enhancing endothelin-1 release from endothelial cells and consequent activation of the endothelin-1 pathway (65). In addition, oxidative stress contributes to vessel thickening as it activates the production of transforming growth factor-β1 (TGF-β1), vascular endothelial growth factor (VEGF), fibroblast growth factor-2 (FGF-2) and platelet-derived growth factor (PDGF) (62). Macrophages, ECs, PASMCs and fibroblasts are sources of ROS and oxidative stress in PAH as nicotinamide adenine dinucleotide phosphate (NADPH) oxidases are found in those cells (66, 67). The NADPH oxidases 1-5 play an important role in lung vasculature by increasing ROS generation which contributes to vascular dysfunction and in turn can lead to further ROS production (62). Other sources of ROS in PAH are the xanthine oxidase (XO) and nitric oxide synthases. During disease state, mitochondria electron transport complexes can also be disrupted and become a source of ROS. However, NADPH oxidases are accepted as the major source of ROS in lung vasculature and are thought to regulate the other sources (64).

ROS production and oxidative stress contribute significantly to normal tissue damage after exposure to ionizing radiation. Radiolysis of water leads to the production of free radicals with very short half-lives immediately after irradiation in the extracellular environment (36, 37). In addition, the redox system contributes to radical production in the time frame of hours after irradiation and can last for years (36). Like in PAH, upregulation of enzymes such as NADPH oxidases play a major role in ROS production. These enzymes have effects on mitochondrial function leading to further ROS production (36) in leukocytes and macrophages but also e.g., in fibroblasts (68). Oxidative stress and ROS in turn, leads to DNA damage which can be followed by apoptosis, but can also contribute to senescence. These processes lead to the secretion of inflammatory cytokines and can therefore contribute to normal tissue damage. As discussed above, these mechanisms are also known to contribute to vascular remodeling in lung tissue which can lead to PAH induction and progression.

### Hypoxia

The relation between hypoxia and PAH is very well established (69). Hypoxia plays a major role in several animal models of PAH, either as chronic exposure to hypoxia alone or in combination with other components e.g., Sugen, a VEGFR-2 antagonist (63). In chronic hypoxic models, where mainly rats but also other animals are exposed to 10-12% oxygen for several days or weeks, pulmonary artery remodeling as well as remodeling of smaller vessels occurs and RV hypertrophy is observed (64). In combination with a single Sugen injection, more severe PAH is developed (63). The combination with hypoxia enhances the effects of overexpression of IL-6 in the lung and leads to a more severe PAH in a rat model (51). In patients, hypoxia may not be a primary reason to develop PAH, but hypoxia contributes to the risk of developing PAH, especially in people with preexisting lung pathologies (70). However, as shown in the animal models, hypoxia can act as prerequisite for the induction of PAH or, in chronic hypoxia be the main inducer of PAH (63, 64).

It has been shown that ionizing radiation can induce hypoxia in the lungs (71). Vujaskovic et al. (71) investigated hypoxia in a rat model after hemithorax-irradiation with 28Gy and found moderate levels of hypoxia in the lungs 6 weeks after irradiation and more severe levels after 6 months. Fleckenstein et al. (72) showed reduced perfusion of the lung early after irradiation in the same model system accompanied by hypoxia, oxidative stress, and enhanced cytokine production. Radiation-induced hypoxia in lung tissue is mainly thought to be related to lung fibrosis; however, vascular remodeling is also shown as a result (72). Hypoxia occurring after irradiation in lung tissue can contribute to an environment which favors the development of PAH, similar to that described for hypoxic animal models of PAH. Furthermore, hypoxia can contribute to ROS formation and activation of other previously discussed mechanisms which contribute to PAH initiation and development.

## Possible interventions

**Figure 3:**
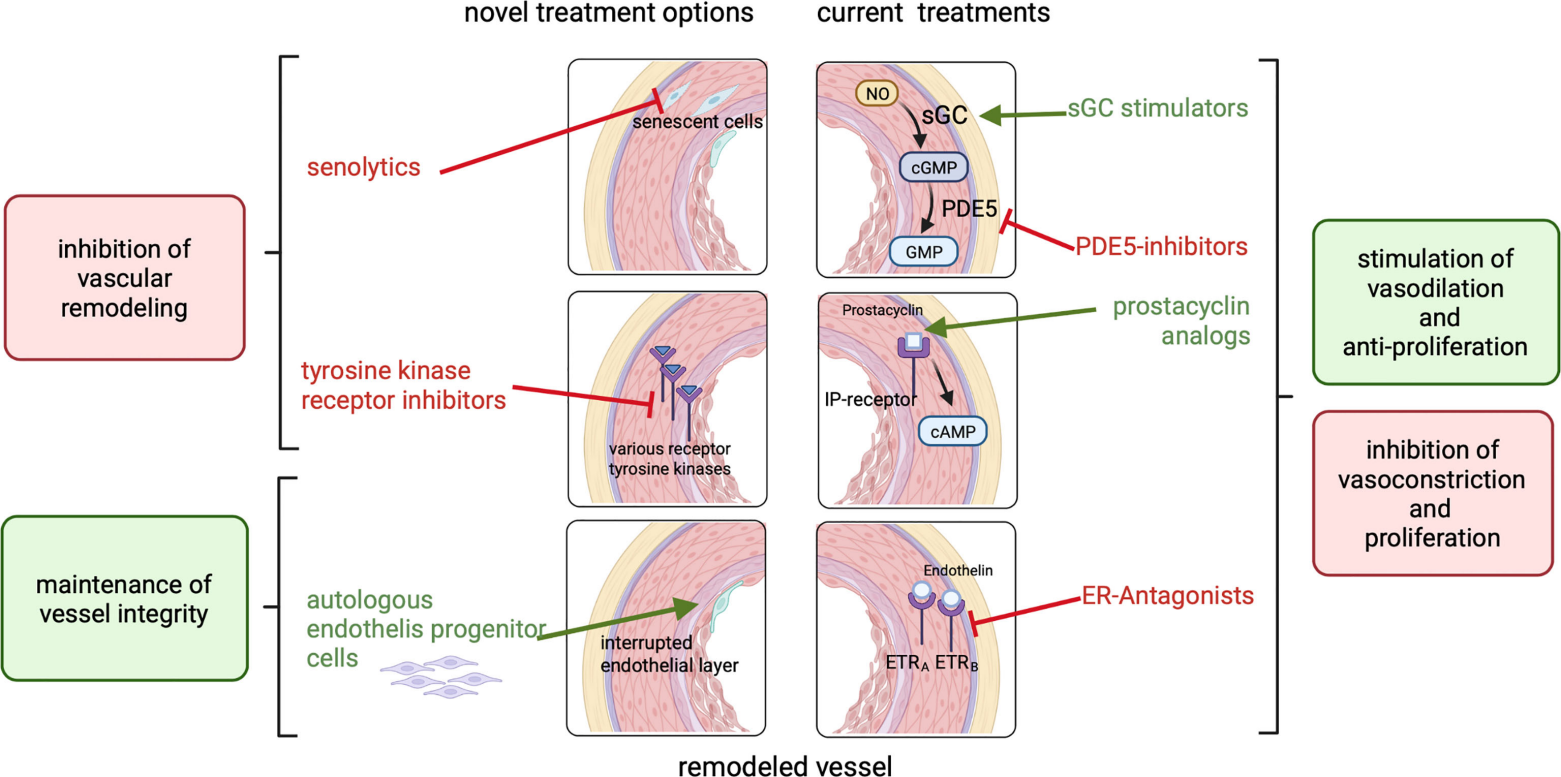
Currently approved treatments for PAH (right side) include ER-antagonists, prostacyclin analogs, PDE5-inhibitors and sGC stimulators which inhibit vasoconstriction and proliferation or act vasodilative and anti-proliferative to reduce blood pressure and inhibit vascular remodeling. Potential novel treatment options (left side) like senolytics or tyrosine kinase receptor inhibitors inhibit vascular remodeling. The treatment with patient-derived endothelial progenitor cells can maintain vessel integrity.

Even if described as limited, treatments for PAH are available in cardiology practice for non-oncological patients. Fourteen FDA approved drugs treating PAH are on the market (73). New drugs, targeting other pathways and mechanisms in PAH are under development and some of them have already been tested in clinical trials (73). As summarized in the previous sections, there are many similarities in the mechanisms leading to pulmonary arterial hypertension and those involved in radiation-induced vascular damage. In line with this, we suggest that treatment options which are available for PAH may potentially also be able to counteract initiation and progression of PAH-like features induced by irradiation. This suggests that the effectiveness of these drugs could be investigated preclinically and subsequently clinically to potentially yield new interventions to specifically prevent or treat pulmonary vascular damage. Therefore, in the following sub-sections current drugs and current developments in the cardiology field that may be of interest for the reduction or prevention of cardiopulmonary damage due to irradiation will be discussed (**Figure 3**).

### Approved drugs for PAH treatment

Approved drugs for PAH can be classified into 4 classes: prostacyclin analogs, endothelin-receptor antagonists (ERAs), phosphodiesterase type 5 (PDE-5) inhibitors and soluble guanylate cyclase (sGC) stimulators. The latter target 3 pathways: excess endothelin (ET) activity, abnormal nitric oxide (NO) activity, and prostacyclin (PGI2) deficiency (73). A combination of an endothelin receptor antagonist and a phosphodiesterase 5 inhibitor is currently widely accepted as standard of care therapy (74). These include, for example, the PDE-5 inhibitors Sildenafil or Tadalafil in combination with Bosentan, Macitentan or Ambrisentan. The available treatments mainly focus on vasodilation and not primarily on the reduction of vascular remodeling (75). However, it has also been shown that vascular remodeling is reduced after treatment with these drugs. The similarities in the mechanisms and events leading to vascular remodeling suggests that the treatment with these medications or a combination of those could potentially be a tool to counteract or prevent the development of PAH in radiotherapy patients and should be further explored.

### New treatment options for PAH

Besides the described routinely used medications, other medical approaches targeting different mechanisms are currently under development for PAH patients. These were recently reviewed in e.g., Sommer er al (76). and Condon et al. (73). For example, modulating the BMP signaling, targeting sexual hormone related pathways or other hormonal pathways are considered as promising tools for PAH treatment. However, these mechanisms may not play a role after irradiation. Here we aim to summarize treatment options which are a potential option for radiotherapy patients and therefore target mechanisms in which parallels were identified in the previous sections.

Altered growth factor signaling is involved in the characteristic hyperproliferation and apoptosis-resistance of endothelial and smooth muscle cells in vascular remodeling of pulmonary arteries in PAH as well as in radiation-induced vascular remodeling. As this signaling involves tyrosine kinase receptors, the use of tyrosine kinase receptor inhibitors is currently tested. An oral tyrosine kinase receptor inhibitor, matinib mesylate, showed promising results in terms of hemodynamic improvement in a stage 3 clinical trial, but had significant side effects (77). Therefore, another clinical trial which aims to find a tolerable dose and treatment regime is currently recruiting (78). In addition, a more specific tyrosine kinase inhibitor binding to PDGF receptors is currently being tested (73).

Another promising approach is the autologous treatment with patient-derived endothelial progenitor cells which are transfected with the endothelial nitric oxide synthase gene. Nitric oxide is a potent vasodilator which is normally synthesized by the (eNOS) and released from endothelial cells. In PAH, as well as after irradiation, the loss of endothelial cells is an initial step of vascular remodeling. In addition, in PAH a reduction of eNOS expression has been shown in the remaining endothelial cells, leading to low levels of NO. Several studies have shown that administration of EPCs restores endothelial function due to vessel repair and promotes angiogenesis by releasing proangiogenic factors (79, 80), while eNOS-transfected cells improved hemodynamic and RV function (81, 82). For PAH patients, this approach is currently tested in a clinical trial. Because the loss of endothelial function represents one of the main steps in vascular remodeling, this treatment approach could be considered for radiotherapy patients. Further investigation would be necessary to check whether eNOS expression in endothelial cells is also reduced after irradiation and treatment with eNOS transfected cells would lead to an additional improvement.

As previously discussed, senescence is another relevant mechanism in both PAH development and after irradiation exposure. Van der Feen (43) showed that a switch from a proliferative to a senescence phenotype in vascular endothelial cells is related to the loss of reversibility in a PAH model. The use of senolytics such as ABT263 proved that removal of senescent cells can facilitate the reversal of haemodynamic changes, as well as structural changes which are present in severe PAH. Although not yet tested clinically, senolytics are currently considered as a potential treatment to counteract adverse effect of normal tissue irradiation (83, 84). Treatment with senolytics after thoracic irradiation may be a promising tool to counteract vascular remodeling and consequent PAH. In addition, the data from van der Feen (43) indicate that reversal of already present PAH is possible if senescence cells are eliminated. Beside the removal of senescent cells, the avoidance of senescence could also be a strategy to reduce side effects after irradiation. Experiments performed at very high dose rates (FLASH irradiation) have shown reduced normal tissue toxicity compared to conventional irradiation (85, 86). Interestingly, the reduced damage to normal tissue is at least in part due to reduced senescence induction, but this requires further investigation. Therefore, modulating the dose rate can be a strategy to reduce radiation induced side effects, accompanied by a reduction of senescence which will also reduce vascular remodeling. In addition, it has been shown that FLASH irradiation also reduces DNA damage in normal tissue which can also contribute to reduce normal tissue effects.

## Discussion

We summarized evidence that loss of cardiopulmonary function due to vascular remodeling similar to that in pulmonary arterial hypertension may play an important role as a side effect of thoracic radiotherapy and we discussed possible novel interventions.

To this end we first showed that there is evidence in preclinical experiments for the occurrence of vascular remodeling in the lungs and consequent right ventricular effects similar to those in pulmonary arterial hypertension after exposure to ionizing irradiation. Next, we established various parallels in the mechanisms playing a role in the induction and development of PAH, and those induced after irradiation. Furthermore, we summarized classical as well as novel treatment options which are used in cardiology praxis to treat PAH and could potentially be used for radiotherapy patients.

This raises the question of what is needed in order to use the knowledge on treatment options of PAH and implement it in the standard pre and/or post-treatment care of radiotherapy patients.

First, in radiotherapy practice, vascular remodeling leading to PAH has not yet been recognized as a possible side effect of radiotherapy. More insights into the effects of irradiation on lung vasculature and the right heart are needed to prove that the preclinically observed effects also occur in patients. Several patient studies show that overall survival is related to lung and/or heart dose (87–89)while some cases of death could not be related to known cardiopulmonary side effects after irradiation, such as pneumonitis or fibrosis (87). This suggests that unrecognized side effects e.g. vascular remodelling leading to PAH-like symptoms, may be involved. However, up to now, the follow up on radiotherapy patients is restricted to classical endpoints of RILT and RIHT, such as fibrosis or lung inflammation. Detection of early PAH-related symptoms in patients requires different diagnostics in the routine follow-ups of radiotherapy patients. For example, echocardiography to check for RV performance and a more specific blood diagnostic checking for specific markers is needed to detect PAH-related changes. The CLARIFY study is the first study investigating more specific PAH related endpoints in radiotherapy patients (90). In this large prospective cohort study lung and esophageal cancer patients receiving thoracic radiotherapy are included. Echocardiography, cardiac MRI and the detection of blood biomarkers is performed to gain more insights into changes potentially related to PAH.

Besides establishing that PAH also occurs in radiotherapy patients, proof of concept is needed to show that interventions are also effective if PAH is induced by radiation. For this, further preclinical studies are needed to test medications approved for PAH in an animal model developing radiation-induced vascular remodeling. There are only very few studies testing e.g., a PDE-5 inhibitor like Tadalafil or Sildenafil to counteract irradiation-induced effects on the lung or the heart (91, 92), and these studies do not address effects on vascular remodeling but instead, for example, check for changes in miRNA levels. Information on the effect of those interventions on vascular remodeling and endpoints such as RV performance is required to assess the potential of these known interventions for PAH for testing in radiotherapy patients.

However, besides the parallels, there are also differences between patients developing radiation-induced pulmonary vascular remodeling and PAH patients. The most obvious difference is that radiotherapy patients receive their treatment because they have cancer. Therefore, when considering any interventions or medication to treat radiation induced PAH, it is important to verify that this does not lead to tumor protection. However, some medications used for the treatment of PAH, such as different endothelin inhibitors and antibodies (93)or PDE-5 inhibitors like Sildenafil (94–96) have been suggested as potential treatment option for cancer. Overexpression of PDE-5 has been reported for lung and breast cancer. Sildenafil is pre-clinical but is thought to not only enhance the sensitivity of different tumor cells to chemotherapeutic agents and reversing multidrug resistance, but also show anti-cancer effects itself (97). The endothelin-axis and especially ET-1 and its receptor are known to be hyperactivated and overexpressed in many malignancies and play a pro-hyperproliferative and pro-survival role (98). Clinical trials to prove this hypothesis are still lacking, but everal trials aiming to answer this question are ongoing (97–99). For other treatment options, effects on the tumor itself need to be considered as well.

Interestingly, lung cancer patients are at increased risk of cardiopulmonary comorbidities (100). In fact, there is evidence that pulmonary hypertension occurs with a higher incidence in those patients (101). For lung cancer, as well as for PAH, similar risk factors related to life-style, such as smoking and being overweight have been identified (102, 103) in addition to genetic predispositions. These are also factors which have to be considered in future approaches to prevent or treat irradiation-induced vascular remodeling.

## Conclusion

There is preclinical evidence that radiation-induced vascular remodeling in the lungs can cause a reduction in right ventricular function. Recognizing similarities between vascular remodeling after irradiation and in PAH and the target cells and mechanisms involved, can open novel avenues for pharmacological prevention or treatment of this severe side-effect of radiotherapy for intra-thoracic tumors.

## Author contributions

JW wrote the first draft of the manuscript and designed the figures. RPC and PvL provided critical feedback and proofread the manuscript. All authors contributed to the article and approved the submitted version.

## Funding

JW and PvL received funding from the Dutch Cancer Society under grant number 12134 (AVERT) and 11349 (CLARIFY).

## Conflict of interest

The authors declare that the research was conducted in the absence of any commercial or financial relationships that could be construed as a potential conflict of interest.

## Publisher’s note

All claims expressed in this article are solely those of the authors and do not necessarily represent those of their affiliated organizations, or those of the publisher, the editors and the reviewers. Any product that may be evaluated in this article, or claim that may be made by its manufacturer, is not guaranteed or endorsed by the publisher.
